# The Microenvironment in Barrett’s Esophagus Tissue Is Characterized by High *FOXP3* and *RALDH2* Levels

**DOI:** 10.3389/fimmu.2018.01375

**Published:** 2018-06-18

**Authors:** Alexandra Lind, Peter D. Siersema, Johannes G. Kusters, Tanja Konijn, Reina E. Mebius, Leo Koenderman

**Affiliations:** ^1^Laboratory of Translational Immunology, Department of Respiratory Medicine, University Medical Center Utrecht, Utrecht, Netherlands; ^2^Department of Gastroenterology and Hepatology, University Medical Center Utrecht, Utrecht, Netherlands; ^3^Department of Medical Microbiology, University Medical Center Utrecht, Utrecht, Netherlands; ^4^Department of Molecular Cell Biology and Immunology, VU University Medical Center, Amsterdam, Netherlands

**Keywords:** Barrett’s esophagus, retinoic acid, *RALDH2*, duodenum, reflux esophagitis, *FOXP3*

## Abstract

Metaplasia in Barrett’s esophagus (BE) is characterized by the transition of squamous epithelium into intestinal-type columnar epithelium. The immune response in BE shares many similarities with the response found in the gut, which is different from the response found in a normal-looking esophagus. Here, we investigated the role of the genes associated with the retinoic acid (RA) pathway in BE, as RA is important not only in shaping the gut’s immune response but also in the induction of metaplasia *in vitro*. mRNA was isolated from esophageal and duodenal biopsies from BE (*n* = 14), reflux esophagitis patients (*n* = 9), and controls (*n* = 12). cDNA was made and qPCR was performed. The expression of RALDH1, CYP26A1, MAdCAM1 were similar for both the BE and duodenum, but different when compared to squamous esophageal epithelium. BE was characterized by a higher expression of RALDH2 and FOXP3, compared to the duodenum. In BE, RALDH2 correlated with expression of the myeloid dendritic cell-specific genes: CD11c and CD1c. Also, RALDH2 expression correlated with RAR-β and FOXP3. Hierarchical clustering on the expression of multiple relevant genes demonstrated that BE, duodenum, and SQ tissues are clustered as three different groups. The differential expression of RA-specific genes and dendritic cell (DC)-subsets indicates that BE resembles duodenal tissue. The higher expression of RALDH2 and FOXP3 in BE points at a mechanism associated with a possible anti-inflammatory microenvironment. This aberrant immune regulation might contribute to the altered tissue and immune responses found in BE.

## Introduction

Barrett’s esophagus (BE) is characterized by a metaplasia from a multi-layered squamous epithelium into an intestinal-like, single-layered cylinder epithelium (1). Patients with BE have an incidence rate of around 1 in 200 per patient-year to develop esophageal adenocarcinoma (EAC) (1). The incidence of EAC continues to increase, and it is currently the fastest rising malignancy in the Western World (2).

Recently, it was shown that the immune cells found in BE and duodenal tissues are characterized by the presence of very similar T-cell phenotypes and a comparable expression of adhesion molecules, which is different from healthy esophageal tissue (3). However, little is known about the underlying mechanism mediating this aberrant immune response in BE tissue. Retinoic acid (RA) plays a key role in immune cell differentiation and an important role in metaplasia and oncogenesis by inducing both pro-oncogenic and anti-oncogenic effects (4–6). If expression levels of RA-pathway-specific genes are similar in BE and duodenal tissue, this would lead to a similar immune microenvironment.

Retinoic acid also plays an important role in the expression of integrins and chemokine receptors in intestinal tissue, thereby steering homing of leukocytes to their homeostatic tissue sites (5). Aberrant RA-metabolism in BE might, therefore, redirect leukocytes to BE tissue that normally would home exclusively to gut tissue. Chang et al. have shown that there are enhanced levels of RA in BE tissue, but the relation between RA and tissue immune response was not investigated. As such, the primary cellular source of RA and its influence on development of Barrett’s adenocarcinoma remain unknown (7).

Chang’s and others findings were supported by experiments *in vitro* showing that RA was able to induce intestinal metaplasia (IM) in squamous esophageal tissue (7, 8). The current study was designed to determine the presence of enzymes that are important in RA synthesis in esophageal tissues from controls and BE patients (9, 10). As dendritic cells (DCs) are an important source of RA, the expression of various DC-markers was also measured (11).

Aim of the study was to investigate the expression of RA-related and DC-specific genes in duodenal and esophageal tissues from controls and BE patients.

## Materials and Methods

### Characteristics of Patients and Controls

Thirty five patients and controls were included in our study (see Table 1). Of these, 14 patients had BE, as defined by the presence of specialized IM containing goblet cells in at least one biopsy. Tissue biopsies were taken from BE epithelium (*n* = 10), duodenal tissue (*n* = 12), and squamous esophageal tissue (*n* = 10). Nine patients had reflux esophagitis (RE), which was characterized by gastroesophageal reflux complaints and fulfilled the endoscopic diagnostic criteria according to the Los Angeles classification (12). From RE patients, tissue was taken from the inflamed and non-inflamed squamous esophageal epithelium. 12 unrelated, healthy controls were selected from patients who underwent upper endoscopy for upper gastrointestinal symptoms other than gastroesophageal reflux disease (GERD), and had no previous history of GERD or immune-associated disorders, such as celiac disease, and no history of duodenal diseases. Symptoms of both controls and RE/BE patients were evaluated by a standardized questionnaire, which needed to be negative for GERD symptoms (13). From five controls duodenal biopsies were taken, which were used for mRNA isolation. Demographic data of patients and controls are summarized in Table 1. Figure S1B in Supplementary Material shows immunohistochemical staining of BE, SQ, and DUO biopsies, used in this study for mRNA isolation.

**Table 1:** Patient characteristics.

	Barrett’s esophagus patients	Controls	Reflux esophagitis
Number of patients	14	12	9
Mean age (±SD)	53 ± 12	58 ± 14	59 ± 14
Gender (% males)	50	25	67
Presence of low grade dysplasia	7%	–	–
PPI use (%)	100	42	89
Hiatal Hernia (%)	86	8	22

The Medical Ethical Committee of the University Medical Center Utrecht approved this study, and written informed consent was obtained from all patients and controls.

### Isolation of Peripheral Blood Mononuclear Cells (PBMCs) and Expansion of Lymphocytes

Blood was obtained from healthy volunteers at the donor service of the Utrecht University Medical Center and anticoagulated with 0.4% (w/v) trisodium citrate (pH 7.4). PBMCs were isolated as described in the literature (14).

### *Ex Vivo* Expansion of T-Cells

Expansion of T-cells from biopsies was performed essentially using methods described in the literature (15).

### Analysis by Flow Cytometry

The immunophenotyping of lymphocytes was performed on collagenated biopsies and on day 14 of the *ex vivo* expansion (3) (Figure S1A in Supplementary Material).

Cells (0.5 × 10^3^–10^5^) were washed with PBS2+ and subsequently incubated for 30 min on ice with directly labeled antibodies (Table 2) according to manufacturer’s instructions. After washing with PBS2+, the cells were analyzed by FACS essentially as described in the literature (3). Cell populations positive for CD4, CD8, CD49d (α4), and β7 were identified by testing the specific antibodies together with the appropriate isotype control on PBMCs. Isotype controls could not be included in the *ex vivo* cultured cells’ experiments due to the small number of cells available.

**Table 2:** Antibodies for FACS staining.

mAb CD3 (T-cell)-FITC	clone sk7, 1:20	BD Biosciences, Bedford, MA, USA
CD3-PE	clone sk7, 1:20	BD Biosciences
CD8 (cytotoxic T-cell)	clone SK1, 1:100	BD Biosciences
CD8-PerCP	clone SK1, 1:20	BD Biosciences
CD4 (T-helper cell)-PerCP	clone L200, 1:20	BD Pharmingen, San Diego, CA, USA
CD49d (α4-integrin)-FITC	clone BU49, 1:20	EuroBiosciences, Friesoythe, Germany
β7 (integrin)-PerCP/Cy5.5	clone FIB27, 1:25	BioLegend, CA, USA

### RNA Isolation and cDNA Synthesis

RNA was purified from esophageal biopsies using the RNeasy Mini Kit (Qiagen, Valencia, CA, USA), according to the manufacturer’s instructions. For cDNA synthesis, 1 µg total RNA was transcribed into cDNA with the cDNA transcription reagents (Bio-Rad, Hercules, CA, USA) using oligo (dT) and random primers according to manufacturer’s instructions.

### Real-Time (RT) PCR: cDNA Analysis

Amplification and RT detection of PCR products with SYBR green was performed using the MyiQ RT PCR detection system (Bio-Rad, Hercules, CA, USA) under the following conditions: 3 min at 95°C and 40 cycles of 30 s at 95°C, 30 s at 60°C, and 30 s at 72°C. The results represent the relative quantity of mRNA levels normalized to the housekeeping gene GAPDH (glyceraldehyde-3-fosfaat dehydrogenase) and plotted as fold change. The expression level of a gene in a given sample was represented as 2^−ΔCT^, where ΔCT = [CT_(experimental)_] − [CT_(housekeeping)_]. PCR assays were performed in duplicate. Primers excluding those described below, were purchased from Sigma Aldrich, St. Louis, MO, USA (Table S1 in Supplementary Material). Primers for RALDH1 (*Aldh1A1*), RALDH2 (*Aldh1A2*) (retinaldehyde dehydrogenases 1,2), RAR-β (RA receptor-β) ubiquitin C, hprt (hypoxanthine-guanine-fosforibosyl-transferase), and GAPDH (Isogen Life Science, De Meern, The Netherlands; Invitrogen) were designed across exon–intron boundaries using Primer Express software (PE Applied Biosystems, Foster City, CA, USA). RT PCR for RALDH1, 2, and RAR-β was performed on an ABI Prism 7900HT sequence detection system (PE Applied Biosystems). The total volume of the reaction mixture was 10 µl, containing cDNA, 300 nM each primer, and SYBR Green Master Mix (PE Applied Biosystems). The comparative *C*_T_ method (Δ*C*_T_) was used to assess relative changes in mRNA levels between samples.

### Statistical Analysis

All continuous variables were analyzed for significance with one-way analysis of variance non-parametric test, using the Kruskal–Wallis test to compare the groups: BE tissue (BE), duodenum of BE patients (DUO BE), duodenum of controls (DUO C), inflamed (INFL RE) and non-inflamed squamous esophageal tissue of RE patients (SQ RE), BE patients (SQ BE), and controls (SQ C). To identify transcripts that were differentially expressed between various tissue types in BE, patients, RE patients, and controls, we used analysis of variance for non-parametric data (Kruskal–Wallis test) with Benjamini–Yekutieli correction for multiple comparisons, as provided by R software (*R* Foundation for Statistical Computing, Vienna, Austria), with False Discovery Rate (FDR) set to 0.05 (16). *p*-values for comparing the individual groups were obtained by using Mann–Whitney *U* test when comparing independent groups and Wilcoxon signed rank test was used when comparing different tissue types in the same patients (e.g., duodenum tissue and Barrett tissue from BE patients).

Spearman’s correlation coefficients (data were non-parametric) were performed to explore association in mRNA expression of RALDH2 with DC-specific markers in BE. Also, correlation analysis was performed on DC-markers in inflamed tissue from RE patients (INFL RE) and duodenal tissues. Correlations were corrected for multiple testing by Benjamini–Yekutiele method, with FDR set to 0.05. Expression of FOXP3 was not corrected for multiple comparison, because the decision to measure FOXP3 expression was made as a consequence of the differential expression of RA-related genes.

A two-sided *p*-value < 0.05 was considered to be statistically significant. Statistical analyses were conducted using GraphPad Prism 5 (GraphPad Software, La Jolla, CA, USA), SPSS (Statistical Package for Social Sciences, version 22; SPSS Inc., Chicago, IL, USA), and R program.

### Hierarchical Clustering

Hierarchical clustering was conducted using the program OmniViz^®^, version 6.1.2 (BioWisdom Ltd., Maynard, MA, USA).

## Results

### Similar mRNA Expression of Adhesion Molecules in Duodenum and BE

**Figure 1:**
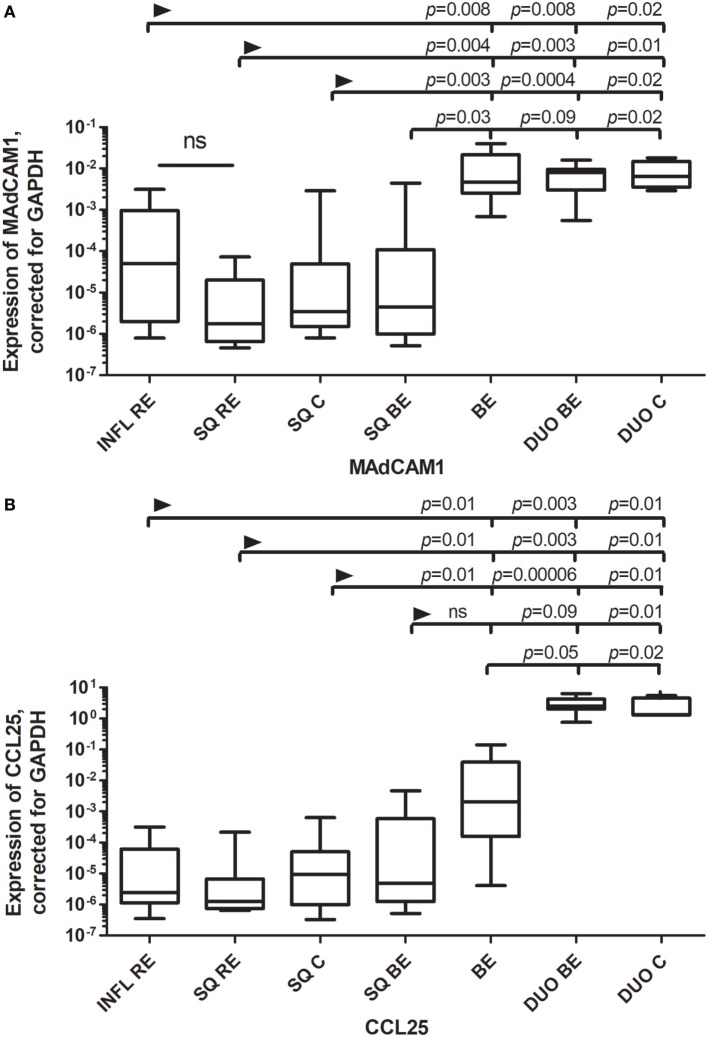
**(A)** Similar expression of MAdCAM1 Barrett’s esophagus (BE) and duodenal tissues. **(B)** Expression of CCL25 in BE is intermediate between squamous esophageal tissue and duodenal tissue. Real-time (RT) PCR was performed on a total of: reflux esophagitis (RE) biopsies (INFL RE, *n* = 9), non-inflamed squamous esophageal epithelium from RE patients (SQ RE, *n* = 9), squamous esophageal epithelium from controls (*n* = 12), squamous esophageal epithelium from BE patients (SQ BE, *n* = 10), BE biopsies from BE patients (BE, *n* = 11), duodenal biopsies from BE patients (DUO BE, *n* = 10), and duodenal biopsies from controls (DUO C, *n* = 5). Expression of MAdCAM1, corrected for GAPDH, 2^−ΔCT^ ± SEM, is represented on the *y*-axis in **(A)**. Expression of CCL25 corrected for GAPDH, 2^−ΔCT^ ± SEM is represented on the *y*-axis in **(B)**. The Kruskal–Wallis test was used to compare all the groups (*p* < 0.0001), *p*-values from comparing individual groups were obtained by using Mann–Whitney *U* test in case of individual groups and Wilcoxon signed rank test for different tissue types from the same patient. Adjustment for multiple comparison was conducted by Benjamini–Yekutieli method.

We determined the expression of mRNA of the endothelial integrin and chemokine receptor ligands MAdCAM1 (mucosal vascular addressin cell adhesion molecule 1), VCAM-1 (vascular cell adhesion protein-1), and the intestinal homing chemokine CCL25 (Chemokine (C-C motif) ligand 25/TECK) (17). Expression of MAdCAM1 was similarly low in squamous esophageal epithelium (SQ) obtained from the different patient groups (RE patients, BE patients, and controls; Figure 1A). Expression of MAdCAM1 in SQ RE, SQ C, and inflamed tissue from RE (INFL RE) was significantly lower compared to the expression of MAdCAM1 in mRNA taken from biopsies of both the duodenum and BE (Figure 1A). Expression of MAdCAM1 in SQ BE was also significantly lower compared to BE and SQ C (Figure 1A). However, expression of MAdCAM1 in SQ BE tended to be lower compared to DUO BE, but the difference did not reach statistical significance (*p* = 0.09) (Figure 1A). Expression of CCL25 was low in SQ for all three groups (RE patients, BE patients, and controls). The expression of CCL25 was markedly enhanced in duodenal tissue of controls and BE patients, which was significantly higher compared to all SQ tissues (Figure 1B) (18). The expression of CCL25 in BE was significantly lower compared to both duodenal tissues, and was significantly higher compared to squamous esophageal tissues in RE patients and controls (Figure 1B). It was, however, not-significantly higher compared to SQ BE (Figure 1B).

**Figure 2:**
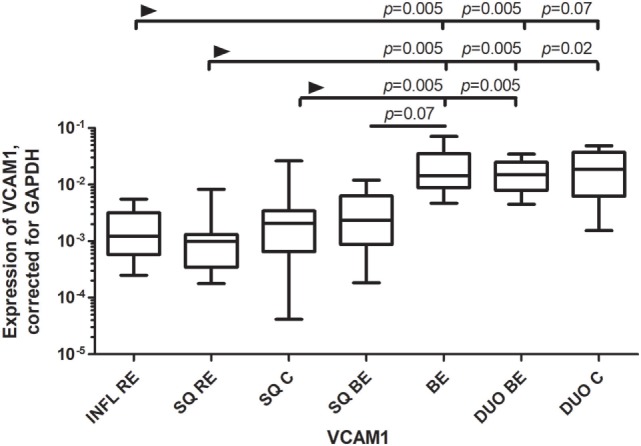
Similar expression of VCAM-1 in Barrett’s esophagus (BE) and duodenal tissues. Figure 2 represents expression of VCAM-1 on the *y*-axis, corrected for GAPDH, 2^−ΔCT^ ± SEM. The Kruskal–Wallis test was used to compare the groups. The Kruskal–Wallis test was used to compare all the groups (*p* < 0.0001), *p*-values from comparing individual groups were obtained by using Mann–Whitney *U* test in case of individual groups and Wilcoxon signed rank test for different tissue types from the same patient. Adjustment for multiple comparison was conducted by Benjamini–Yekutieli method.

VCAM-1, a ligand for VLA-4 (very late antigen 4; α4β1) and α4β7 (19), is usually upregulated under inflammatory conditions (19). However, the expression of VCAM-1 was found to be similar in BE and duodenum, and this expression was significantly higher or almost reached significance compared to most SQ groups of RE/BE patients and controls (Figure 2).

### High Expression of RALDH1 mRNA in BE Tissue and Duodenal Tissue of BE and Controls; High Expression of RAR-β mRNA in BE

**Figure 3:**
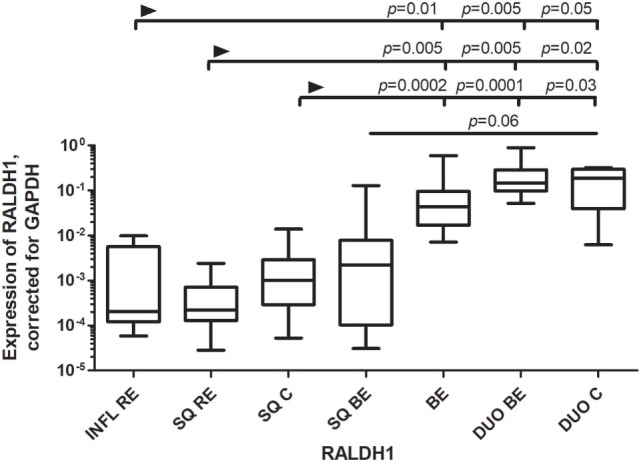
Low expression of RALDH1 in squamous esophageal tissues and similarly high expression of RALDH1 in Barrett’s esophagus (BE) and duodenal tissues. Figure depicts expression of RALDH1 by real-time-PCR performed on a total of: inflamed tissue from reflux esophagitis (RE) (INFL RE, *n* = 9), non-inflamed squamous esophageal epithelium from RE patients (SQ RE, *n* = 9), squamous esophageal epithelium from controls (*n* = 12), squamous esophageal epithelium from BE patients (SQ BE, *n* = 10), BE tissue from BE patients (BE, *n* = 11), duodenal biopsies from BE patients (DUO BE, *n* = 10), and duodenal biopsies from controls (DUO C, *n* = 5). The Kruskal–Wallis test was used to compare the groups (*p* < 0.0001). *p*-values from comparing individual groups were obtained by using Mann–Whitney *U* test in case of individual groups and Wilcoxon signed rank test for different tissue types from the same patient. Adjustment for multiple comparison was conducted by Benjamini–Yekutieli method.

RALDH1 is normally expressed by epithelial cells, including gut epithelial cells, which can produce RA in small amounts (20). RALDH1 expression was similar in BE and duodenum. RALDH1 expression in DUO BE was significantly higher compared to inflamed (INFL RE) and non-inflamed SQ from RE patients and controls (Figure 3). RALDH1 expression in duodenum of controls (DUO C) and BE was significantly higher compared to INFL RE, SQ RE, and SQ C (Figure 3). Expression of RAR-β in SQ of all groups was similar (data not shown). BE was characterized by highest RAR-β expression compared to other groups (data not shown).

### High Expression of CYP26A1 mRNA in RE

**Figure 4:**
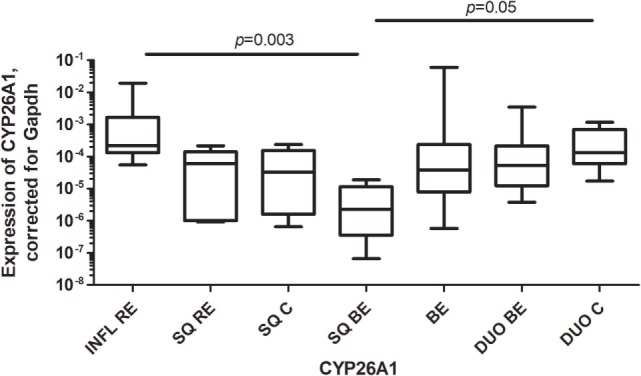
High expression of CYP26A1 in inflamed reflux esophagitis tissue. Figure depicts expression of CYP26A1 by real-time-PCR, corrected for GAPDH, 2^−ΔCT^ ± SEM (*p* = 0.0005). *p*-values from comparing individual groups were obtained by using Mann–Whitney *U* test in case of individual groups and Wilcoxon signed rank test for different tissue types from the same patient. Adjustment for multiple comparison was conducted by Benjamini–Yekutieli method.

CYP26A1, a catabolizing enzyme of RA, was highly expressed in inflamed tissue of RE (INFL RE) when compared to SQ of BE patients (Figure 4). Expression of CYP26A1 in duodenal tissue of controls (DUO C) was significantly higher compared to SQ of BE patients (Figure 4). Expression of CYP26A1 was similar in BE for both the intestinal tissues of BE and duodenum (Figure 4).

### High Expression of RALDH2 mRNA in BE Tissue Compared to Duodenum and SQ

**Figure 5:**
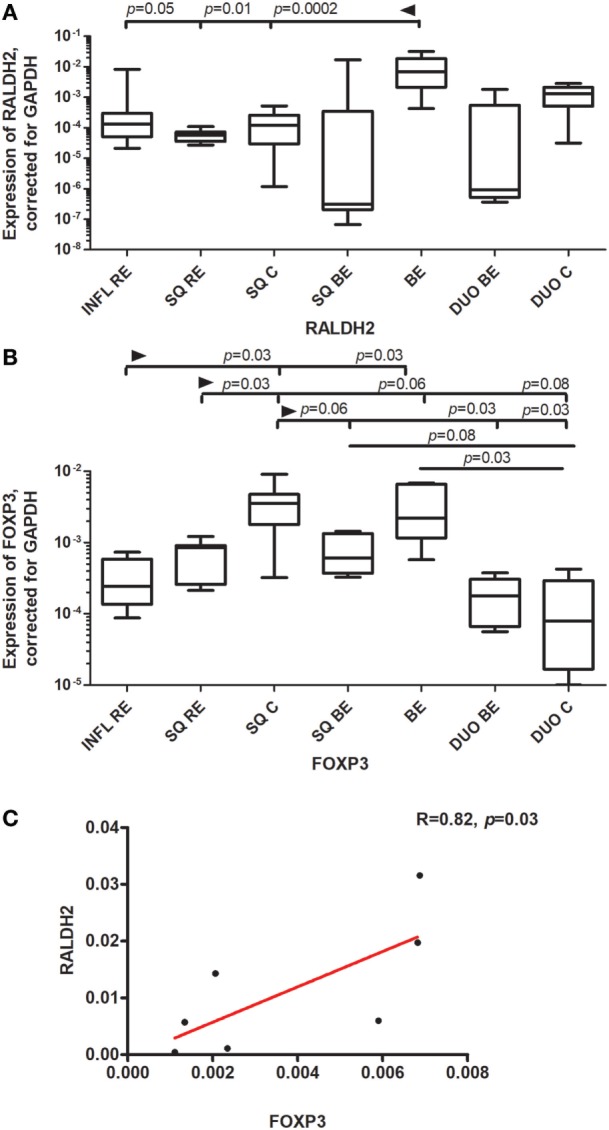
High expression of RALDH2 and FOXP3 in Barrett’s esophagus (BE) tissue. Panel **(A)** depicts expression of RALDH2 by real-time (RT)-PCR performed on a total of: inflamed reflux esophagitis (RE) biopsies (INFL RE, *n* = 9), non-inflamed squamous esophageal epithelium from RE patients (SQ RE, *n* = 9), squamous esophageal epithelium from controls (SQ C. *n* = 12), squamous esophageal epithelium from BE patients (SQ BE, *n* = 10), BE biopsies (BE, *n* = 11), duodenal biopsies from BE patients (DUO BE, *n* = 10), and duodenal biopsies from controls (DUO C, *n* = 5). Panel **(B)** depicts expression of FOXP3 by RT-PCR performed on a total of: inflamed RE biopsies (INFL RE, *n* = 6), non-inflamed squamous esophageal epithelium from RE patients (SQ RE, *n* = 7), squamous esophageal epithelium from controls (SQ C, *n* = 12), squamous esophageal epithelium from BE patients (SQ BE, *n* = 7), BE biopsies (BE, *n* = 8), duodenal biopsies from BE patients (DUO BE, *n* = 5), and duodenal biopsies from controls (DUO C, *n* = 5). In panels **(A,B)** data were analyzed, using the Kruskal–Wallis test (RALDH2: *p* < 0.0001, FOXP3: *p* < 0.0001). RALDH2 expression and FOXP3 expression was corrected was corrected for GAPDH, 2^−ΔCT^ ± SEM. *p*-values from comparing individual groups were obtained by using Mann–Whitney *U* test in case of individual groups, and Wilcoxon signed rank test for different tissue types from the same patient. Adjustment for multiple comparison was conducted by Benjamini–Yekutieli method. In panel **(C)**, correlation and linear regression (red line) between expression of FOXP3 and RALDH2 in BE biopsies are shown, *R* (correlation coefficient) = 0.82, *p* = 0.03. The Spearman correlation was conducted as there was no normal distribution of data.

RALDH2 is highly expressed in DCs and plays a crucial role in the synthesis of RA in mucosal immune tissues (21). The expression of RALDH2 was significantly higher in BE compared to squamous esophageal tissues from RE patients and controls (INFL RE, SQ RE, SQ C) (Figure 5A). The expression of RALDH2 in BE was higher than in duodenum of BE patients, but it did not reach significance when corrected for multiple testing (Figure 5A). The expression of RALDH2 in INFL RE and non-inflamed SQ (SQ from RE/BE patients and controls) was not-significantly different from each other (Figure 5A).

### High Expression of FOXP3 mRNA in BE and Its Correlation With the Expression of RALDH2 in BE

FOXP3 (forkhead box P3) is a master regulator in the development and function of regulatory T-cells (22). FOXP3 was highly expressed in SQ from controls (SQ C). This expression was statistically significantly higher compared to inflamed (INFL RE) (*p* = 0.03) and non-inflamed SQ from RE patients (SQ RE) (*p* = 0.03) (Figure 5B). Expression of FOXP3 was also high in BE, which was significantly higher compared to duodenal tissue from controls (*p* = 0.03) (Figure 5B). Further, FOXP3 expression correlated strongly with the expression of RALDH2 in BE tissue (*r*_s_ = 0.82, *p* = 0.03, Figure 5C).

### Correlation Between mRNA Expression of RALDH2 and Myeloid Dendritic Cell (mDC) Markers in BE

To look into which cell type could be responsible for high RALDH2 levels in BE, correlation analysis on RALDH2 and DC-related mRNA was performed. mRNA RALDH2 expression in BE biopsies correlated significantly (*p* = 0.02) with the mRNA expression of CD11c, a mDC marker (Table 3, upper section) (23). In addition, in BE, RALDH2 correlated with CD1c, which is also an mDC marker (Table 3). RALDH2 expression levels also correlated with RAR-β in BE tissue (*p* = 0.07), suggesting that the increased expression of RALDH2 in BE biopsies is associated with a higher expression of RAR-β (Table 3).

**Table 3:** Correlations between the relative expression of mRNA in different tissue types.

Barrett’s esophagus (BE)	RALDH2 vs. CD11c	RALDH2 vs. CD1c	RALDH2 vs. RAR-β			
*r*_s_^a^	0.88	0.7	0.79			
*p*-Value*	0.02	0.08	0.04			

**INFL reflux esophagitis (RE)**	**CD11c vs. CD1a**	**CD11c vs. CD1c**	**CD11c vs. DC-sign**	**CD1c vs. CD1a**	**CD1c vs. DC-sign**	**CD1a vs. DC-sign**

*r*_s_	0.95	0.92	0.88	0.88	0.95	0.82
*p*-Value	0.001	0.0028	0.006	0.006	0.001	0.03

**Duodenum**	**CD11c vs. CD83**	**D83 vs. DC-sign**	**CD11c vs. CD123**			

*r*_s_	0.74	0.74	0.76			
*p*-Value	0.004	0.004	0.004			

*Upper section: correlations between RALDH2 with CD11c, CD1c, and RAR-β in BE tissue; middle section: correlations of mRNA expression levels in INFL RE; lower section: correlations in duodenal tissue. Correlations were adjusted for multiple testing by Benjamini–Yekutieli method*.

*^a^Correlation coefficient*.

*Significant if p < 0.05.

### mRNA Expression of DC-Specific Markers in Squamous Esophageal Tissue and Intestinal Tissue Was Associated With Distinct T-Cell Phenotypes

**Figure 6:**
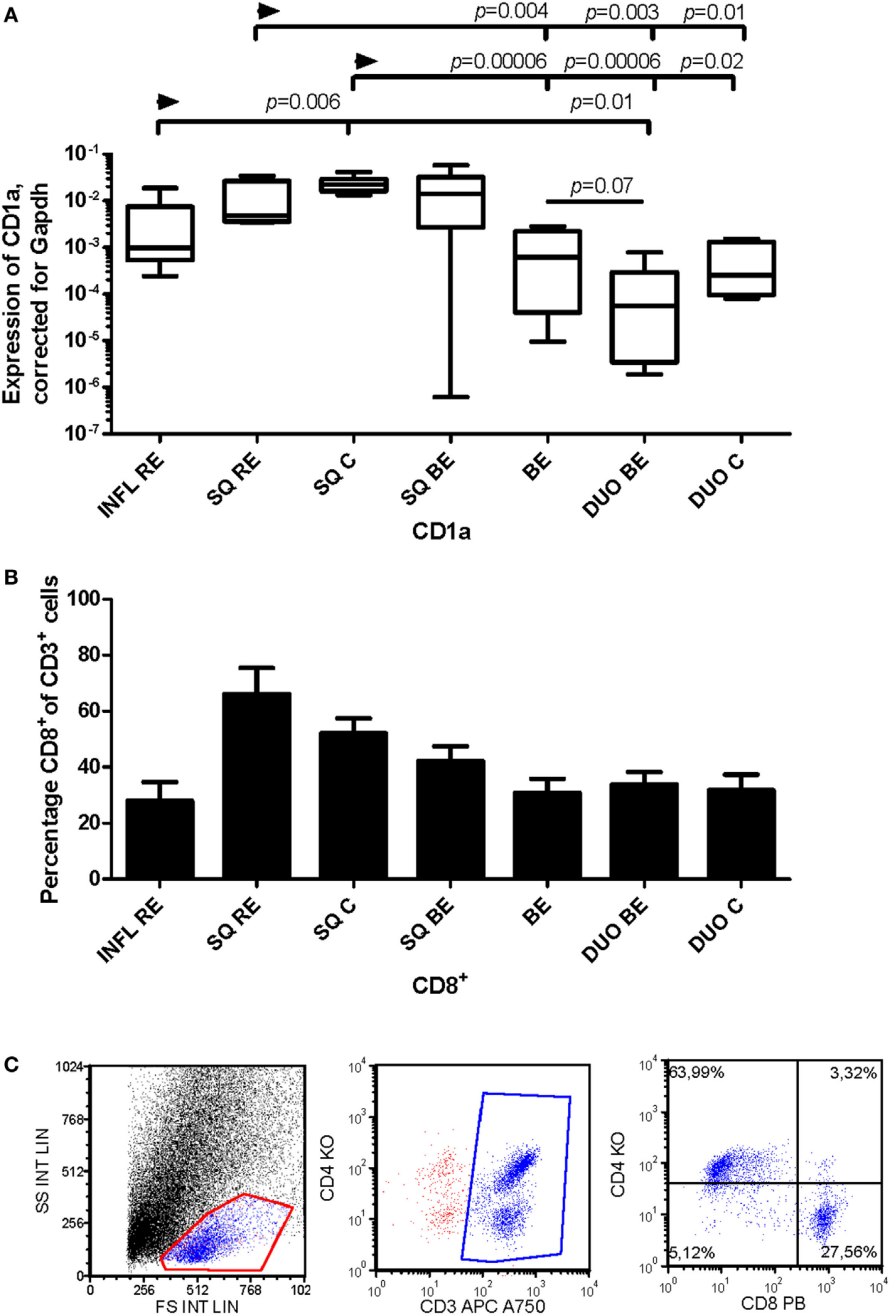
Lower expression of CD1a in Barrett’s esophagus (BE) is associated with lower numbers of CD3^+^CD8^+^-cells in tissue. Panel **(A)** depicts expression of CD1a by RT-PCR performed on a total of: reflux esophagitis (RE) biopsies from inflamed squamous tissue (INFL RE, *n* = 9), non-inflamed squamous esophageal epithelium from RE patients (SQ RE, *n* = 9), squamous esophageal epithelium from controls (SQ C, *n* = 12), squamous esophageal epithelium from BE patients (SQ BE, *n* = 10), BE biopsies (BE, *n* = 11), duodenal biopsies from BE patients (DUO BE, *n* = 10), and duodenal biopsies from controls (DUO C, *n* = 5). CD1a expression was corrected for GAPDH, 2^−ΔCT^ ± SEM. Panel **(B)** represents the percentage of CD3^+^CD8^+^-cells from *ex vivo* cultures of 7 biopsies from inflamed RE tissue (INFL RE), 6 non-inflamed squamous esophageal biopsies from RE patients (SQ RE), 39 controls (SQ C), 17 squamous esophageal biopsies from BE patients (SQ BE), 19 BE biopsies (BE), 15 duodenal biopsies from BE patients (DUO BE), and 11 duodenal biopsies from controls (DUO C). Each bar represents the mean value ± SEM of the percentage of CD3^+^CD8^+^-cells in the CD3^+^ population, determined by flow cytometry. Data were analyzed using the Kruskal–Wallis test (CD1a: *p* < 0.0001, CD3^+^CD8^+^: *p* = 0.0002). *p*-values from comparing individual groups were obtained by using Mann–Whitney *U* test in case of individual groups, and Wilcoxon signed rank test for different tissue types from the same patient. Adjustment for multiple comparison was conducted by Benjamini–Yekutieli method. **(C)** Dot plot of a CD3^+^, CD4^+^, and CD8^+^ staining on lymphocytes from *ex vivo* culture of duodenal tissue from BE patient. Cells were gated first on a forward sideward scatter plot and for CD3.

CD1a is a marker of mDCs and Langerhans cells (24) and is also present on immature DCs (25). CD1a^+^ DCs can enhance cytotoxic CD8^+^-T-cell responses (26). There was a higher expression of CD1a mRNA in non-inflamed squamous esophageal tissues (SQ RE, SQ C, and SQ BE) (Figure 6A) compared to BE and duodenal tissues (Figure 6A). In INFL RE, there was a significantly lower expression of CD1a compared to SQ from controls, *p* = 0.006 (Figure 6A). The duodenum of BE patients contained lower amounts of CD1a mRNA (*p* = 0.07) compared to BE tissue (Figure 6A). The high expression of CD1a corresponded with a higher percentage of CD8^+^-cells in non-inflamed SQ (Figure 6B). Figure 6C shows a representative flow cytometry experiment.

**Figure 7:**
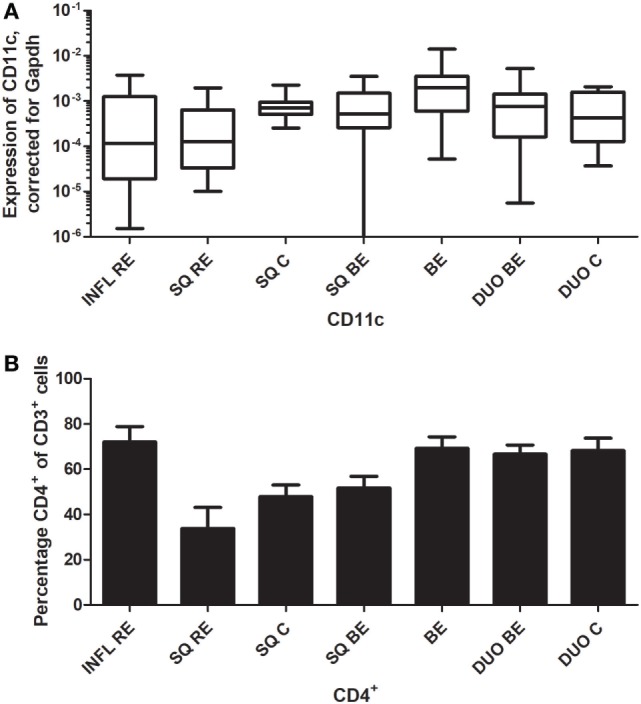
Similar CD11c expression in Barrett’s esophagus (BE) and duodenal tissues coincides with higher numbers of CD3^+^CD4^+^-cells. Panel **(A)** depicts expression of CD11c by real-time-PCR performed on a total of: biopsies from inflamed tissue in reflux esophagitis (RE) patients (INFL RE, *n* = 9), non-inflamed squamous esophageal epithelium from RE patients (SQ RE, *n* = 9), squamous esophageal epithelium from controls (SQ C, *n* = 12), squamous esophageal epithelium from BE patients (SQ BE, *n* = 10), BE biopsies (BE, *n* = 11), duodenal biopsies from BE patients (DUO BE, *n* = 10), and duodenal biopsies from controls (DUO C, *n* = 5). CD11c expression was corrected for GAPDH, 2^−ΔCT^ ± SEM. Panel **(B)** represent the percentage of CD3^+^CD4^+^-cells from *ex vivo* cultures of 7 biopsies from inflamed RE tissue (INFL RE), 6 non-inflamed squamous esophageal biopsies from RE patients (SQ RE), 39 controls (SQ C), 17 squamous esophageal biopsies from BE patients (SQ BE), 19 BE biopsies (BE), 15 duodenal biopsies from BE patients (DUO BE), and 11 duodenal biopsies from controls (DUO C). Each bar represents the mean value ± SEM of the percentage of CD3^+^CD4^+^-cells in the CD3^+^-population, determined by flow cytometry. Data were analyzed using the Kruskal–Wallis test (CD11c: *p* = 0.05, CD3^+^CD4^+^: *p* = 0.0002). *p*-Values from comparing individual groups were obtained by using Mann–Whitney *U* test in case of individual groups, and Wilcoxon signed rank test for different tissue types from the same patient. Adjustment for multiple comparison was conducted by Benjamini–Yekutieli method.

CD11c is a classic marker for DCs, specifically for mDCs (27). These cells are important in activating CD4^+^-T-cells (27). C11c expression was not different in inflamed (INFL RE) tissue compared to non-inflamed SQ from RE/BE patients and controls (Figure 7A). While BE tissue was the highest in expression of mRNA of CD11c, it was not statistically significant (Figure 7A). In BE and duodenal tissue, there were more CD4^+^-cells found compared to non-inflamed SQ (Figure 7B).

### Correlations Between Expression of DC-Specific mRNA and mRNA Expression of RA Pathway Markers in Biopsies From Non-Inflamed SQ, RE Tissue, and Duodenum

In non-inflamed SQ from RE, BE patients, and controls, there were no strong correlations (*r*_s_ > 0.7 or *r*_s_ < −0.7) between DC-specific markers at the mRNA level and expression of proteins involved in the RA pathway.

In inflamed SQ of RE patients, there were strong correlations found in mRNA expression of different DC markers. CD11c expression correlated strongly with CD1a (*r* = 0.95), CD1c (*r* = 0.92), and DC-sign (DC-specific C-type lectin) (*r* = 0.95) mRNA expression (Table 3, middle section). CD1a mRNA expression levels also correlated with those of CD1c and DC-sign (Table 3, middle section). In addition, CD1c correlated with DC-sign in RE. In the duodenum, CD11c correlated with CD83 and CD123 (Table 3, lower section). Correlations of mRNA expression levels of DC markers in the duodenum are summarized in Table 3, lower section. These correlations were not found in BE and SQ (results not shown). There was no correlation between RALDH2 and DC-specific mRNA in the duodenum.

### Multidimensional Analysis by Hierarchical Clustering

**Figure 8:**
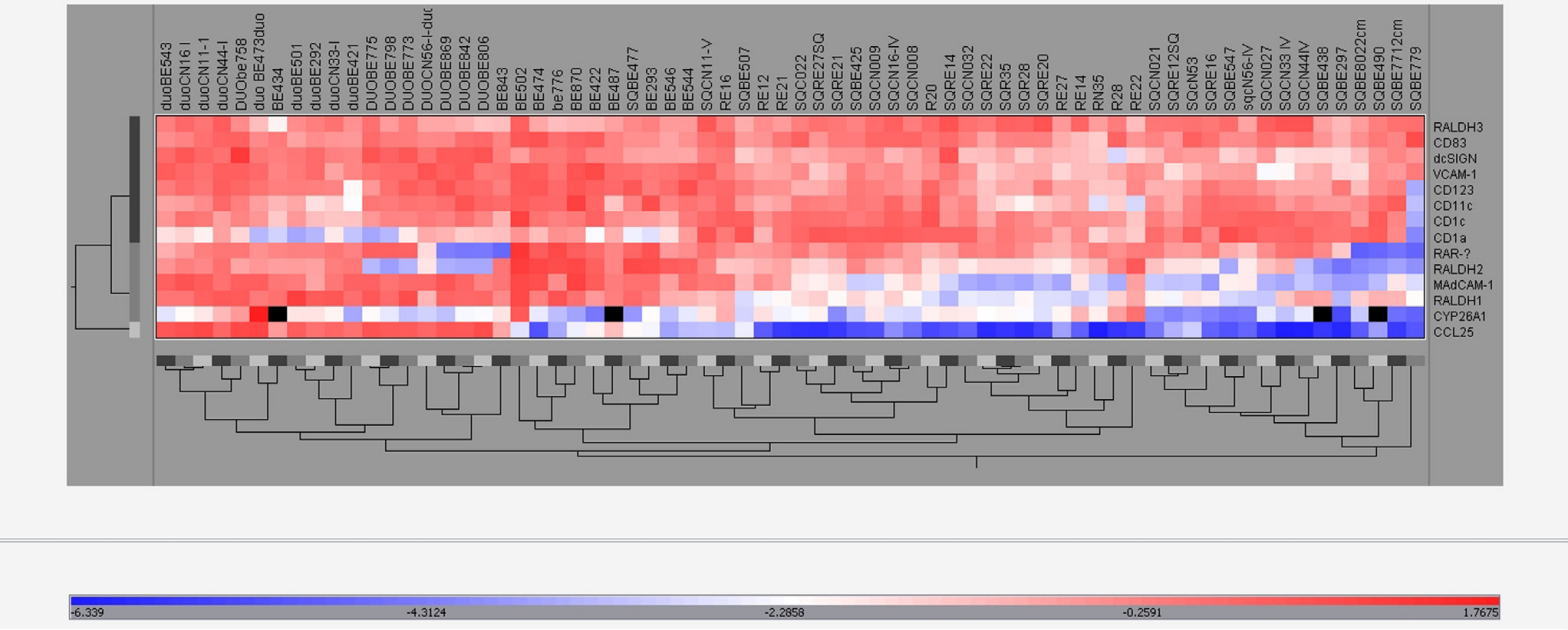
Hierarchical clustering of real-time-PCR data. The expression of all different mRNAs of all groups (BE, BE tissue; SQBE, squamous esophageal tissue from BE patients; DUOBE, duodenal tissue from BE patients; RE, inflamed esophageal tissue from RE patients; SQRE, non-inflamed squamous esophageal tissue from RE patients; SQC, esophageal tissue from controls; DUOC, duodenal tissue from controls) were clustered.

To substantiate the coordinated mRNA expression of the tested RA-associated genes in BE, SQ, and duodenum, the data were analyzed and visualized by hierarchical clustering. Hierarchical cluster analysis of all RA-related and DC-specific mRNA expression levels indicates that BE, duodenum, and SQ were clustered separately (Figure 8).

## Discussion

The study by Lind et al. has provided evidence that, in terms of tissue response, the immune microenvironment in BE is more like duodenal tissue than inflamed esophageal SQ (3). These findings led to the hypothesis that the immune response in BE tissue resembled the more homeostatic immune response found in duodenal tissue rather than an inflammatory response. In our study, this hypothesis was tested in more detail by measuring the expression of RA-pathway-associated genes and DC-specific genes, as RA is important in the immune homeostasis in gut tissue (11). In addition, RA has been implicated in tissue metaplasia such as seen in BE (10).

We found that mRNA expression of RALDH1, MAdCAM1, and VCAM-1 were comparable in BE and duodenal tissue, and markedly different in squamous esophageal epithelium. This is in line with our hypothesis that BE is characterized by an intestinal rather than an inflammatory microenvironment (Figures 1–3) (3).

There are, however, also clear differences between the duodenum and BE; e.g., the expression of CCL25 is very high in duodenal tissue and significantly lower in BE tissue (Figure 1B). Apparently, BE tissue is not completely differentiated into a gut-like environment, possibly explaining the partial phenotype. This conclusion is further supported by analysis of RAR-β expression, which is seen as an *in vivo* readout of RA activity (28). RAR-β mRNA expression is high in BE; this correlates with RALDH2 expression, supporting the concept that RALDH2 activity is associated with a high RA production in the tissue (Figure 5; Table 3). CYP26A1, an enzyme that catabolyzes RA, was found to be lower in SQ BE (significantly, *p* < 0.0005) and tended to be lower BE (not-significantly), compared to inflamed tissue of RE (Figure 4). CYP26A1 is elevated in Barrett’s adenocarcinoma, and can cause proliferation of epithelial cells when overexpressed (29). Zolfaghari et al. found that inflammation can influence RA-metabolism by abrogating the induction of CYP26A1 by RA in the liver. However, we found the opposite: induction of CYP26A1 was highest in inflamed tissue in RE (30). Apparently, the RA-metabolism in esophageal tissue is distinct from systemic RA-metabolism. Yet, lower mRNA expression of RALDH1-2 and RAR-β in SQ, as found in our study, is in line with relatively higher CYP26A1 expression.

Barrett’s esophagus was characterized by the highest expression of RALDH2 (Figure 5A). RALDH2 is highly expressed in DCs, and therefore, the mRNA expression of DC-specific markers was measured and compared with the expression of RALDH2 in the same samples. The correlations between the expression of RALDH2 and CD11c and CD1c implicated that mDCs were associated with high RALDH2 expression (Table 3) and high RA production, as found by Chang et al. (7).

This conclusion was further supported by the findings that mRNA for FOXP3 was markedly enhanced in BE (Figure 5B) and that RALDH2 expression correlated strongly with FOXP3 in this tissue (Figure 5C). The FOXP3 gene in regulatory T-cells has been described to be induced by RA (31, 32). The correlation between RALDH2 and FOXP3 in BE tissue supported the hypothesis that the higher expression of FOXP3 was the result of higher RALDH2 expression, which has also been shown in gut tissue (21). Interestingly, the high expression of FOXP3 supports the hypothesis of an anti-inflammatory, gut-like microenvironment in BE rather than a pro-inflammatory microenvironment. This is contrary to the view that BE is solely an inflammation-driven phenomenon and points to an induction of anti-inflammatory homeostatic responses by aberrant BE tissue in the esophagus (3, 33).

By analyzing DC markers in the duodenum, a correlation was found between the expression CD11c and CD83, as well as between CD83 and DC-sign. These correlations were not seen in BE, suggesting the presence of different DC-phenotypes in the duodenum and BE (Table 3) (34). In BE, CD11c correlated with CD1c and RALDH2, suggesting mDCs, which are rich in RALDH2; whereas in the duodenum, more activated mature DCs were found (34).

No similarities were found between INFL RE and BE. In RE, there were strong correlations between CD1c, CD1a and CD11c, and DC-sign, which implies the presence of a population of monocyte-derived DCs, typically found at sites of inflammation (Table 3, middle section) (35–37). In BE, these correlations were not present, again suggesting that BE was not associated with an ongoing inflammation such as found in inflamed tissue of RE. Expression of dendritic markers varied strongly per patient as seen in Figures 6 and 7. Yet, correlations between the dendritic markers in RE and duodenal had shown high correlation strength (*r* = 0.95), even after correction for multiple comparison.

With respect to DCs (mRNA), our data are in line with our previously published research concerning T-cell phenotypes (3). High CD1a expression in non-inflamed SQ was associated with a high percentage of CD8^+^-cells. The CD1a^+^-DCs are important in activation of these cytotoxic T-cells (Figure 6) (26). High CD11c expression in BE correlated with a higher percentage of CD4^+^-T-cells. CD11c^+^-DCs are important for the activation of CD4^+^-T-cells (Figure 7) (27). This implies that different DC-subsets are seen in BE tissue and squamous esophageal tissue, which might lead to differential activation of distinctive T-cell phenotypes.

To visualize the effect of RA, which is produced mainly by DCs *in vivo*, we performed *in vitro* experiments (32). By adding RA to the PBMCs, we showed that α4 and β7 were upregulated on T-cells, validating the finding that RA induced intestinal integrins on lymphocytes (Figure S2 in Supplementary Material).

The data mentioned above support the hypothesis that the immune homeostasis in BE tissue resembles duodenal tissue more than esophageal tissue. However, most of the data were based on the analysis of single markers. To strengthen this hypothesis, all of the markers that were used in this study were evaluated using cluster analysis (Figure 8). Hierarchical clustering of expression of genes showed that tissues of BE, duodenum, and squamous esophageal tissues are clustered as three different groups (Figure 8). This is in line with our hypothesis that SQ tissue and BE are distinct tissue types. Furthermore, we have shown that BE and duodenum share common characteristics, and these are found to be associated with high activity of the RA-pathway (7, 29). These data also provide a mechanism for RA-regulated homeostatic homing of immune cells to BE tissue. Unfortunately, as biopsies were small and we were restricted in the number of biopsies we could obtain, it was not possible to perform experiments where we could see which cells were responsible for differential gene expression.

In conclusion, the immune microenvironment of BE shares many similarities with that of duodenal tissue, but also has its own special features. This suggests (3) that BE is characterized by the presence of tolerogenic RALDH2^+^DCs and regulatory T-cells, pointing to a non-inflammatory environment in BE tissue. Thus, our findings suggest that aberrant RA-mediated responses, rather than inflammation, are important in pathogenesis of BE. Therefore, application of anti-inflammatory drugs as treatment for BE remains questionable, as there are no clear signs of inflammation in BE.

## Ethics Statement

This study was carried out in accordance with the recommendations of Medical Ethical Committee of the University Medical Center Utrecht with written informed consent from all subjects. All subjects gave written informed consent in accordance with the Declaration of Helsinki. The protocol was approved by the Medical Ethical Committee of the University Medical Center Utrecht.

## Author Contributions

AL, PS, LK, and HK worked on the formulation of the hypothesis and design of the study (including obtaining approval of the medical ethical committee); AL collected patient material, performed experiments (on *ex vivo* expansion of lymphocytes and flow cytometry), performed the statistical analysis, and wrote the first draft of the manuscript; AL and TK: performed PCR experiments; LK supervised AL in experiments and data analysis; AL, LK, PS, HK, and RM contributed to interpretation of the data. LK, PS, and HK contributed to manuscript revision. All authors read and approved the submitted version.

## Conflict of Interest Statement

The authors declare that the research was conducted in the absence of any commercial or financial relationships that could be construed as a potential conflict of interest.

## Abbreviations

mDC, myeloid dendritic cell.
