# Supplementary material for: The Microenvironment in Barrett’s Esophagus Tissue Is Characterized by High FOXP3 and RALDH2 Levels
Source: Front Immunol. 2018 Jun 18;9:1375. doi: 10.3389/fimmu.2018.01375 (PMC6015910; doi:10.3389/fimmu.2018.01375)
Supplement: Supplementary file 1 [file Image_1.PDF]

(1) (2) (3)

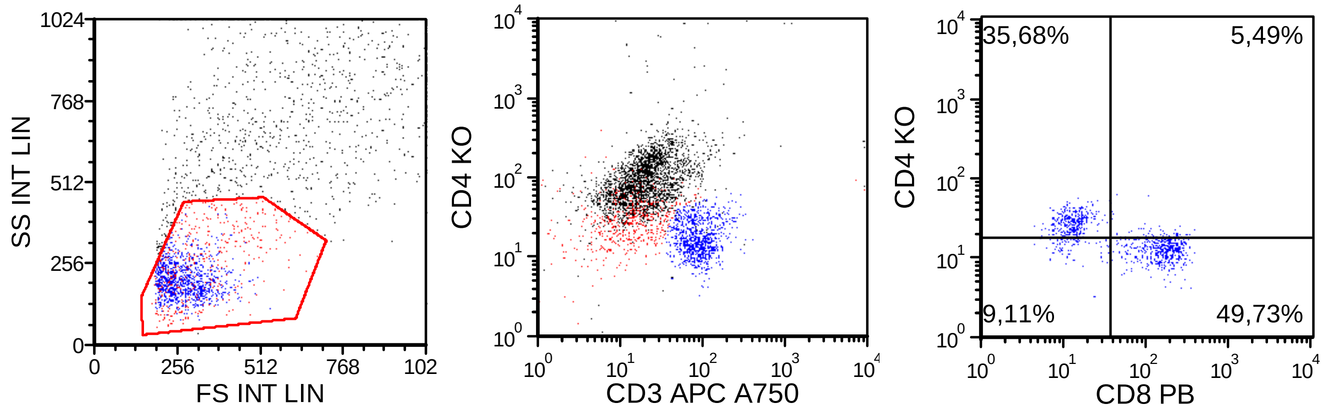

Supplementary Fig. 1 A. CD3, CD4 and CD8 staining on lymphocytes from collegenated biopsy. Biopsy was taken from squamous esophgeal tissue from BE patient. Cells were gated on Forward sideward scatter and also first (1), and as CD3+ cells, second (2). (3) shows CD4 and CD8 stainig on gated cells.

(1)

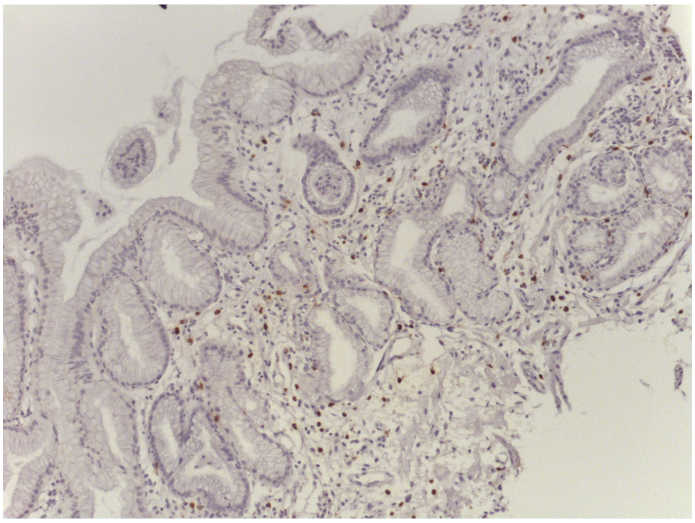

(2)

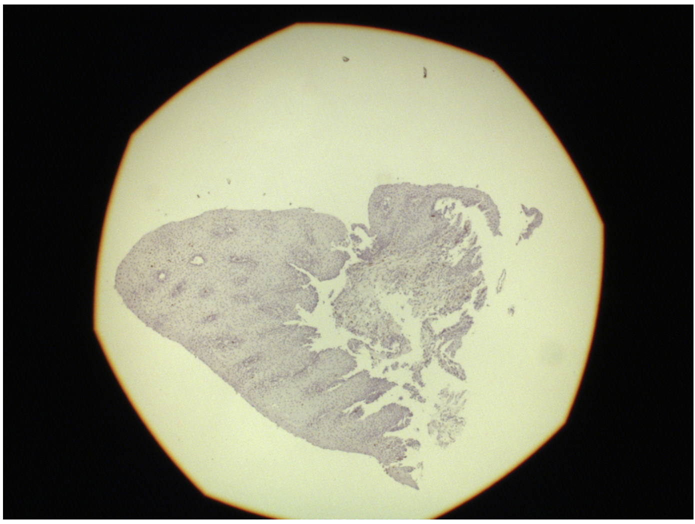

(3)

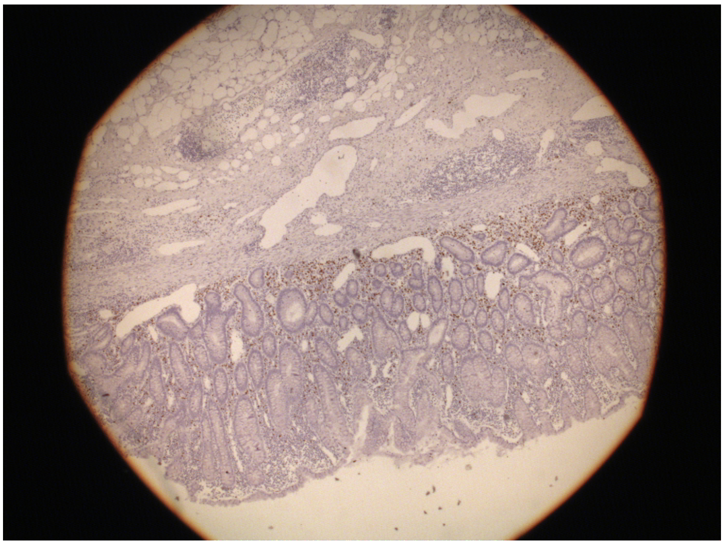

Supplementary Fig. 1 B. Representation of types of tissue used in the study for mRNA isolation(1): Barrett's esophagus tissue, staining with tryptase to identify mast cells. (2): biopsy taken from esophagus with squamous epithelium. (3): biopsy from duodenum, stained with Major Basic Protein (eosinophil staining).
