# Supplementary material for: The Microenvironment in Barrett’s Esophagus Tissue Is Characterized by High FOXP3 and RALDH2 Levels
Source: Front Immunol. 2018 Jun 18;9:1375. doi: 10.3389/fimmu.2018.01375 (PMC6015910; doi:10.3389/fimmu.2018.01375)
Supplement: Supplementary file 2 [file Image_2.PDF]

Supplementary Figure 2

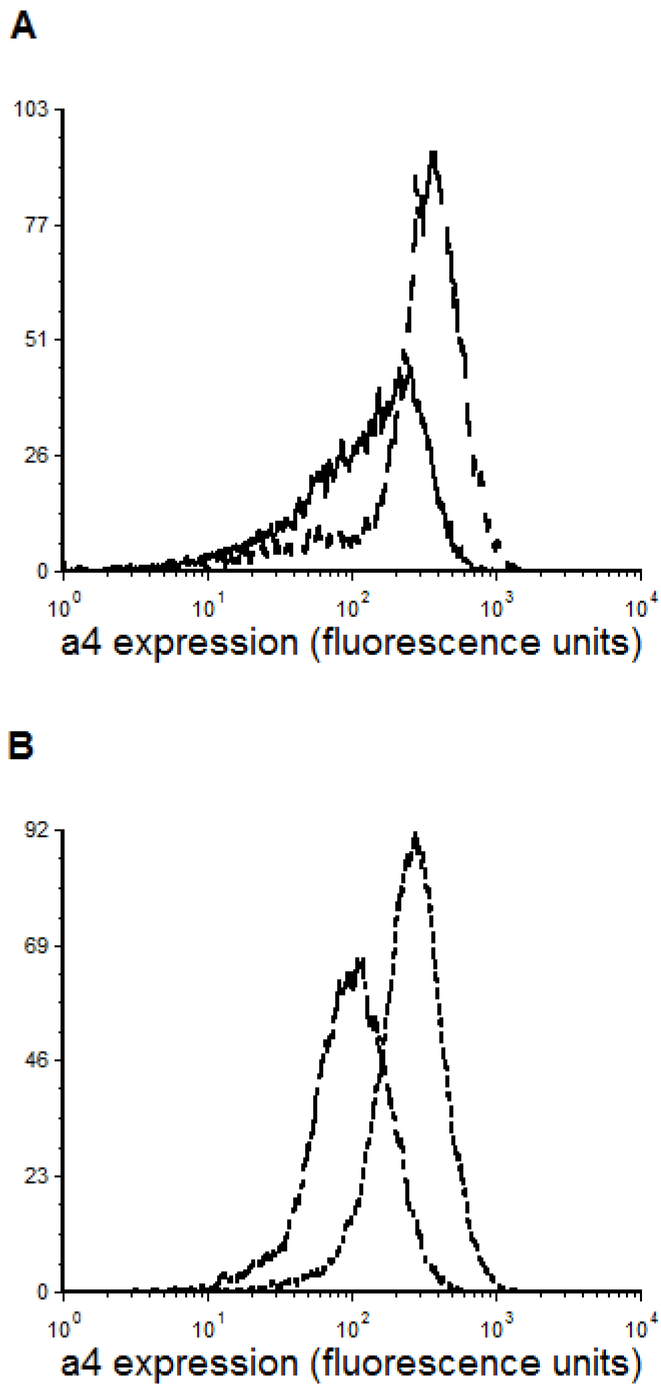

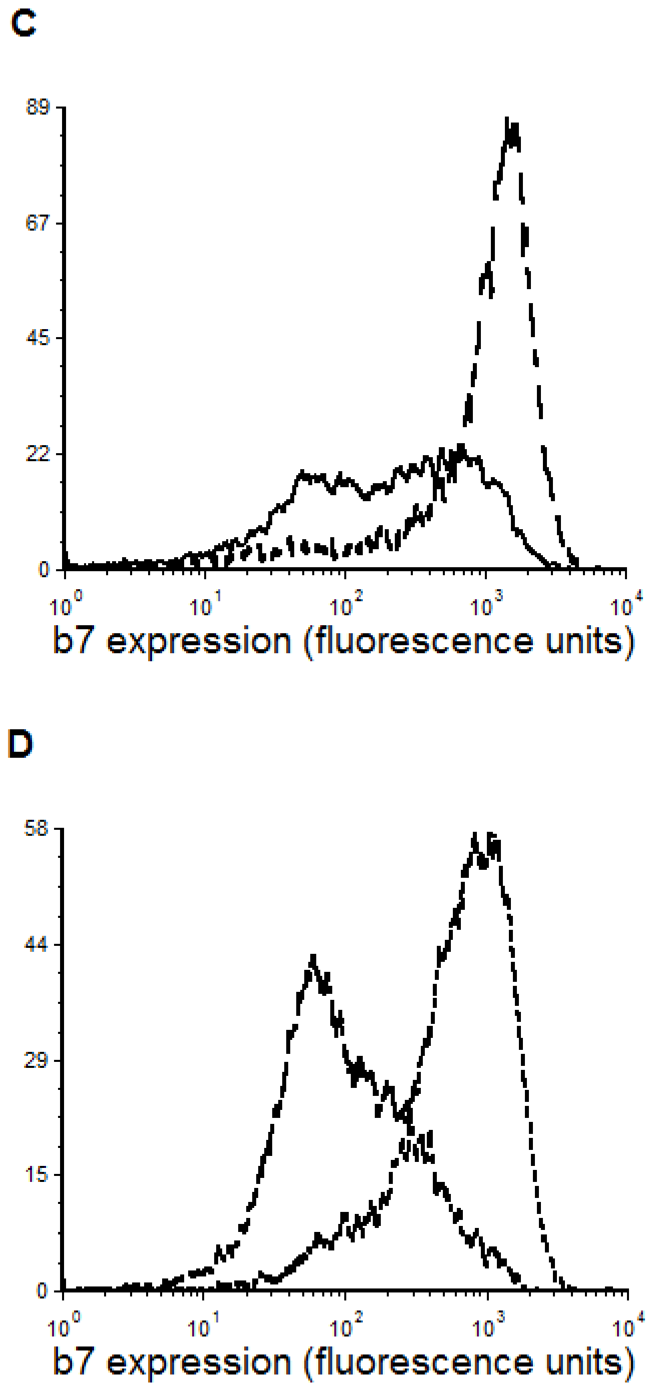

Supplementary Fig. 2 RA upregulates α4 and β7 on lymphocytes (PBMC's incubated in 48-wells plates (Invitrogen)) with IL-2). Expression of α4 (Figures 1 A and 1 B) and β7 (Figures 3C and 3D) in PBMCs with and without the addition of all-trans 20nM Retinoic Acid (Sigma Aldrich, St. Louis, USA). Lymphocytes in Figures B and D were stimulated with CD3 (clone CLB-T3/4.E 16A9, 0.15µg/ml, Sanquin, Amsterdam, The Netherlands)and CD28 (CLB-CD28/1, 15E8, 0.15µg/ml, Sanquin, The Netherlands). The data are representative for 4 additional experiments.
