# Supplementary material for: The Microenvironment in Barrett’s Esophagus Tissue Is Characterized by High FOXP3 and RALDH2 Levels
Source: Front Immunol. 2018 Jun 18;9:1375. doi: 10.3389/fimmu.2018.01375 (PMC6015910; doi:10.3389/fimmu.2018.01375)
Supplement: Supplementary file 3 [file Table_1.doc]

**Supplementary Table 1**. Primers used in PCR reactions.

| **Primer name** | **Sequence** | **Product length** |
| --- | --- | --- |
| CD11c-F | 5’-AGACAGCGTGGTCCAGTATG | 262 |
| CD11c-R | 5’- GAGTCCGGTGAGGTACATGTT |
| CD123-F | 5’- AGGCTCAGCAGTTGACCTG | 233 |
| CD123-R | 5’- GCAGGTCAGATTCTCCGC |
| CD1a-F | 5’- TGAGGGAATTCGTAGATACGCC | 187 |
| CD1a-R | 5’- CATATTCCCAGCCACTGGAT |
| CD1c-F | 5’- CTGCAGTTTCTGCTGCTAGC | 106 |
| CD1c-R | 5’- GGTTGACAAATGAGAAGATCTGGA |
| MAdCAM-1-F | 5’-ACGCAGGGAGAAGTGATCCCAACA | 126 |
| MAdCAM-1-R | 5’-TTTCCAGAGGTGATACGTGGGCAA |
| CCL25-F | 5′-CCATCAGCAGCAGTAAGAGG | 131 |
| CCL25-R | 5′-CTGTAGGGCGACGGTTTTAT |
| VCAM-1-F | 5’- GCTGCTCAGATTGGAGACTCA | 100 |
| VCAM-1-R | 5’- CGCTCAGAGGGCTGTCTATC |
| DC-sign-F | 5′-TCT CCT GGC TCT TTG ACA | 127 |
| DC-sign-R | 5’-ccaccacgatgaatactaca |
| CD83-F | 5’-TCCTGAGCTGCGCCTACAG | 73 |
| CD83-R | 5’-AAGTCCACATCTTCGGAGCAA |
| GAPDH-F | 5’-agaaggctggggctcattt | 136 |
| GAPDH-R | 5’-gaggcattgctgatgatcttg |
| RALDH-1-F | 5’-TTCTTCTCACATGGATATAGACAAAGTAG | 98 |
| RALDH-1-R | 5’-CCCTCTTCAGATTGCTTTTCCCG |
| RALDH-2-F | 5’-AAAGGACTGGGCCGAAGG | 113 |
| RALDH-2-R | 5’-TCTAAAATTTCCTGAACAGGGCC |
| RAR-β-F | 5’-TTCACCTTTGCCAACCAGCTCC | 101 |
| RAR-β-R | 5’-TCAAGGTCCTGGCGGTCTCC |
| CYP26A1-F | 5’- GGAGACCCTTCGACTGAATCC | 91 |
| CYP26A1-R | 5’- GCCCTTGGGAATCTGGTATCC |
| FOXP3-F | 5’-GCGTGGCGTAGGTGAAAG | 83 |
| FOXP3-R | 5’-AAACAGCACATTCCCAGAGTTC |
